# INSAR Special Interest Group Report: Stakeholder Perspectives on Priorities for Future Research on Autism, Sexuality, and Intimate Relationships

**DOI:** 10.1002/aur.2340

**Published:** 2020-06-25

**Authors:** Jeroen Dewinter, Anna I. R. van der Miesen, Laura Graham Holmes

**Affiliations:** ^1^ GGzE Eindhoven The Netherlands; ^2^ Tranzo, Scientific Center for Care and Wellbeing Tilburg University Tilburg The Netherlands; ^3^ Department of Child and Adolescent Psychiatry Center of Expertise on Gender Dysphoria, Amsterdam UMC, VU University Medical Center Amsterdam The Netherlands; ^4^ A. J. Drexel Autism Institute Drexel University Philadelphia Pennsylvania USA

**Keywords:** autism, sexuality, gender identity, community‐based participatory research, quality of life, health, education

## Abstract

The number of empirical studies on sexuality and intimate relationships in autistic people has grown over the last years with the increasing awareness that sexuality and intimate relationships are an important part of life and well‐being for autistic people. Further, expression and enjoyment of sexuality is a fundamental, basic human right. This paper reports on needs for future research in this area based on the input of autistic adults, researchers, and other stakeholders (e.g., parents and professionals). Utilizing the nominal group technique, 65 individuals participated in eight groups in which they brainstormed on research questions they deemed most important. Responses were categorized into themes and ranked according to importance based on the level of priority attributed by participants. Findings suggest that future research should focus on developing ways to support sexual and relationship well‐being and getting a better understanding of sexuality and relationships in autistic people. Also, attention was drawn to the need for studying the influence of stereotypical societal views, and stigma. Finally, the importance of participatory research to include perspectives of autistic people in research and practice was stressed.

**Lay Summary:**

Sexuality and romantic relationships are part of daily life for most people, including autistic people. For this study, groups of autistic people, professionals, and autism researchers discussed which research on autism, sexuality, and relationships is needed and can help autistic adolescents and adults. The group discussions revealed that more research is needed on how to support well‐being relating to romantic relationships and sexuality in autistic people and how the people around them can contribute to this. Therefore, we also need to learn more about how autistic people of all ages and throughout their lives experience sexuality and relationships. Finally, the need for attention to the role of stereotypical ideas and stigma about autism, sexuality, and relationships was pointed out. Attention to the experiences of autistic people can help professionals, researchers, and policy makers to offer and organize attuned support and do relevant research. ***Autism Res** 2020, 13: 1248–1257*. © 2020 The Authors. *Autism Research published by International Society for Autism Research* published by Wiley Periodicals LLC.

## Introduction

Sexuality, an expansive concept that includes a person's behaviors, thoughts, attitudes, attractions, beliefs, identities, and relationships [World Health Organization (WHO), 2006], brings opportunities for interpersonal connection, enhanced well‐being [Diamond & Huebner, 2012], and preventable or reducible emotional, legal, and health‐related risks [Glasier, Gülmezoglu, Schmid, Moreno, & Van Look, 2006; Starrs et al., 2018]. Sexual health, as a state of well‐being toward sexuality, is recognized as a human right [WHO, 2006].

Views on sexuality and autism have changed dramatically over the past four decades and the number of related studies have rapidly increased [Bertilsdotter Rosqvist, 2014; Dewinter, Vermeiren, Vanwesenbeeck, & van Nieuwenhuizen, 2013; Kellaher, 2015; Pecora, Mesibov, & Stokes, 2016]. The autism research discourse has shifted from ignoring sexuality to documenting and preventing problematic outcomes (e.g., socially inappropriate sexual behavior) toward a more holistic focus on sexuality development, gender and sexual diversity, comprehensive sexuality education and romantic relationships as a natural expression of humanity for all persons. Despite this shift, there is limited information about complex issues such as trajectories of sexuality development, healthy and satisfying relationships, and access to appropriate sexual and reproductive health services in autistic people [Dewinter, Vermeiren, Vanwesenbeeck, & van Nieuwenhuizen, 2013; Gougeon, 2010; Hancock, Stokes, & Mesibov, 2017; Kellaher, 2015; van der Miesen, Hurley, & de Vries, 2016]. Advocacy groups, federal advisory committees, and stakeholder focus groups have highlighted the need for further research on autism and sexuality [Halladay et al., 2015; Interagency Autism Coordinating Committee, 2017; Koffer Miller, Mathew, Nonnemacher, & Shea, 2018; Strang et al., 2020; Warner, Parr, & Cusack, 2018]. However, there is currently no systematic, stakeholder‐driven research agenda driving global investment in this area. To address this, a Special Interest Group (SIG) was convened at the 2018 meeting of the International Society for Autism Research (INSAR) to solicit input on key research priorities from autistic people, researchers, clinicians, and other stakeholders.

Stakeholder perspectives have become more central to setting agendas for systematic research into aspects of autism and in health research more broadly [Halladay et al., 2015; Pellicano, Dinsmore, & Charman, 2014; Vivanti et al., 2018; Warner, Parr, & Cusack, 2018]. Stakeholder–researcher collaborations ensure that research addresses questions relevant to served communities and increase the potential that research will ultimately enhance people's lives [Fletcher‐Watson et al., 2018]. The aim of this project is to define a stakeholder‐based set of research priorities to guide future research with the ultimate goal of promoting sexual well‐being, health, and quality of life over the lifespan in autistic people.

## Method: Developing a Stakeholder‐Informed Research Agenda

Participants were recruited from an international research conference and two autism self‐advocacy and support groups in The Netherlands. First, participants were recruited at the May 2018 INSAR Sexuality SIG meeting in Rotterdam, The Netherlands (*n* = 39). SIG attendees sat in five groups ranging from 5 to 12 people and included autistic participants and autism advocates, professionals and trainees from various disciplines, family members, autism researchers, and some identifying with multiple roles (see Table 1). Between June and September 2018, three additional group meetings with autistic adults were convened. Autiroze is a Dutch LGBT+ (lesbian, gay, bisexual, transgender, plus other identities) group for autistic people. Autiroze group members (*n* = 20) in two cities participated during one of their regularly scheduled meetings. Persons on the Autism Spectrum The Netherlands (PAS; *n* = 6) is a self‐advocacy group for autistic adults. Six people responded to our call in the PAS‐newsletter to join a group meeting organized in a local mental health care center. In total, eight small groups of participants from these three settings participated (*n* = 65) including 31 who self‐identified as autistic. No exclusion criteria for participation were applied.

**Table 1:** Participants Characteristics

Role	Nonautistic participants	Autistic participants
Researcher	12	2
Clinician and researcher	9	1
Clinician	6	0
Student	4	1
Family member^a^	3	1
Self‐advocate^b^	0	26

Region	Number of participants
Europe	43
Middle and North America	10
Asia	5
Oceania	2
Unknown	5

^a^ Nonautistic family members included one clinician, one autism advocate, and one student.

^b^ From self‐advocacy or support groups Autiroze and Persons on the Autism Spectrum (PAS) Netherlands.

Research needs and priorities were elicited using the nominal group technique (NGT), which is a structured small‐group method for generating ideas and defining healthcare priorities [Delbecq & Van de Ven, 1971; McMillan et al., 2014; Vella, Goldfrad, Rowan, Bion, & Black, 2000]. All meetings began with a presentation by the authors of an overview of research on autism, sexuality, and relationships, and an explanation of the goals and structure of the meeting. The NGT procedure consisted of three phases [McMillan et al., 2014; Van Breda, 2005]. During Phase 1, participants silently used a report form to generate answers to the following question: “*In your opinion, what are research priorities relating to sexuality and relationships functioning in adolescents and adults with ASD that can add to their well‐being and/or can be of interest to the people around them*.” In Phase 2, participants took turns in their small groups reading aloud research topics that they generated during Phase 1. Participants' ideas were clarified, grouped, and written down by a note taker (one of the authors or collaborating researchers). In Phase 3, participants silently selected the five research topics they believed to be the most important and provided rankings indicating the importance of each topic (5 = “most important” to 1 = “least important”).

**Figure 1:**
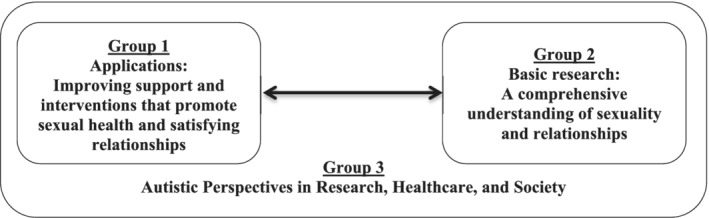
Thematic groupings. All topics prioritized by the participants related to 17 themes, brought together in three thematic groups. The Figure illustrates a meaningful relationship between the groups.

Using the NGT, participants formulated 222 different research questions and topics relating to autism, sexuality, and relationships. These topics were grouped into meaningful categories using inductive thematic analysis. Two of the authors independently developed two lists of themes based on the input of the first five group meetings. Next, the two lists of themes were compared and integrated, resulting in a final list of 17 themes. The information of the next additional three group meetings revealed no new themes, suggesting saturation had been reached. Two authors independently coded all 222 topics. Finally, rankings of the importance of themes were calculated based on the number of topics in a theme, the number of times the topics were selected as a priority by the participants, and by calculating the weighted value attributed to topics within each theme. Based on discussions in the groups and on analysis of the topics, we grouped the 17 themes into three meaningful thematic groupings (see Fig. 1), which are elaborated below.

## Results: Understanding and Supporting Sexuality and Changing Societal Perspectives

First, the three thematic groupings will be described. Then, we present the related themes in more detail. Table 2 shows the ten highest ranked themes for all participants and for autistic participants separately, with example questions from participants for each theme. The main prioritization was based on the values (5–1) attributed by participants. The Table also includes a ranking based on the number of times a theme was selected, independent of the value, and on the number of topics that related to the theme. Due to two sets of tied ranks, the list includes 12 themes. These themes will be described below.

**Table 2:** Highest Ranked Themes^a^

Ranking based on				Themes	Thematic grouping	Definitions	Number of questions related to theme	Example topics/questions
Prioritization within the full sample	Prioritization in the groups with only autistic participants	Number of votes in the full sample	Number of votes in the groups with only autistic participants					
1	2	1	2	How to support healthy and satisfying romantic relationships?	1	Topics relating to finding, keeping, and dealing with yourself and (a) partner(s) in a romantic/sexual relationship	45	How to improve communication skills when in a romantic relationship? How can autistic people be supported in developing authentic relationships without imposing neurotypical norms about how dating/relationships work? How can people learn to manage emotions in a romantic relationship: For example, obsession with partner?
2	3	2	4	How to support and promote sexual well‐being?	1	Topics relating to training and education of knowledge and skills in the autistic individual	33	What are effective strategies for appropriate sex education for autistic women? What is the influence of the environment on perception of which sexual behaviors are accepted and which are inappropriate? How do we accurately measure the impact of parent training and sexuality education on outcomes?
3	1	3	1	How do autistic adolescents and adults experience and navigate sexuality across the lifespan?	2	Topics relating to sexual and relationship experiences and needs in different stages of life	25	What are common trajectories in sexuality development and relationships for autistic people? What is the role of early negative (social) experiences on sexuality and relationships? How do older autistic adults experience sexuality in their lives: What are the positive and negative experiences, successes, and failures?
4	>10^c^	4	>10^c^	How to prevent sexual victimization and sexual offending?	1	Topics relating to the prevention of and care to victims and individuals who have offended	12	How can abuse/exploitation be recognized by people themselves and by others? What are mechanisms behind (unintentional) sexual offending? How can we prepare autistic women to negotiate sexuality without being taken advantage of?
5	8	5	8	How to involve parents, partners, and professionals to support sexual well‐being and relationship satisfaction?	1	Topics relating to involvement, knowledge, attitudes, and skills of caregivers and professionals	16	How can we educate and change parents and stakeholders' attitudes toward developing partnership and sexuality in autistic persons? How best to support professionals how to do this work well? How can professionals best invite autistic people to discuss sexuality and sexual diversity?
6	4	8	3	How does sexual and gender identity develop in autism?	2	Topics relating to identity development	23	What is the interrelationship between formation of different identities (autistic, gender, sexual) and do they have impact on each other? How do autistic people define their sexual identity?
7^d^	6	6	5^d^	What is unique about autistic sexuality and intimacy?	2	Topics relating to specific autism features (e.g., sensory sensitivity) relating to sexuality and relationships	13	What is the influence of information processing characteristics and autistic thinking on sexuality? What the influence of sensory processing characteristics on sexual experience? Is there a connection between interoception and sexual awareness?
7^d^	5	9	5^d^	What works for whom in relationships?	2	Topics relating to types of relationships, characteristics of the relationship, and/or partners	10	How do people experience having a neurotypical versus autistic partner? What are expectations toward romantic relationships? How do autistic people experience different types of relationships? (e.g., both autistic partners, LAT)
9	7	7	5^d^	What are the experiences and needs of LGBT+ identifying autistic individuals?	2	Topics relating to the experiences of autistic LGBT+	7	How do autistic LGBT+ individuals experience concealing or divulging multiple identities? (“double coming out”) How do autistic individuals feel about diversity?
10^d^	9	10	7	Autistic perspectives in research, healthcare, and society	3	Topics relating to societal views, ideas, and stereotypes	10	What are parents, stakeholders, and society's attitudes toward partnership and sexuality of autistic individuals? How does stigma affect dating experiences?
10^d^	>10^c^	>10^c^	10	How do sexuality and relationships develop in autistic individuals with lower levels of intellectual functioning?	2	Topics relating to sexuality and relationships in autistic individuals with intellectual disabilities	6	What are the experiences and needs relating to sexuality and relationships of autistic people with intellectual disabilities? What are development trajectories in sexuality in autistic individuals with an intellectual disability?

^a^ Twelve themes are included in the Table due to tied ranks.

^b^ Thematic groupings are (1) improving support and interventions that promote sexual health and satisfying relationships, (2) comprehensive understanding of sexuality and relationships, and (3) autistic perspectives in research, healthcare, and society.

^c^ Themes were ranked outside of the top ten for group.

^d^ Tied for ranking.

A first thematic grouping was related to *improving support and interventions that promote sexual health and satisfying sexual experiences and relationships*. Themes in this group addressed the need for effective sexuality education and sexual healthcare services, learning how to navigate sexuality and relationships, support for satisfying romantic relationships, preventing sexual victimization and sexual offending, and the importance of involvement, knowledge, attitudes, and skills of parents, other caregivers, and professionals. The second thematic grouping was related to *gaining a comprehensive understanding of sexuality and relationships* in autistic people across childhood, adolescence, and adulthood, with attention to diversity in terms of cultures, intellectual functioning, and diverse gender and sexual identities. Participants stressed the need to understand what is unique about autistic sexuality development, particularly how autism traits influence sexuality development and starting and maintaining different types of relationships. Finally, themes in the third grouping referred to questions about stereotypical ideas regarding autistic sexuality, resultant stigma, and the hope that an improved understanding of autism, sexuality, and relationships can *influence societal views* and challenge these stereotypes and myths about autistic sexuality. Within this grouping, *incorporating autistic perspectives in research and healthcare* was stressed in order to accelerate the process of gaining a comprehensive understanding and applying that knowledge to improve the lives of autistic people and those around them.

### *Group 1: Improving Support and Interventions that Promote Sexual Health and Satisfying Sexual Experiences and Relationships*

Participants identified a need for research on effective supports and interventions that would promote skills that facilitate sexual satisfaction and healthy relationships. Stakeholders acknowledged that romantic partners, friends, parents, siblings, and others close to an autistic person need guidance on how to provide support for healthy sexuality development. Addressing the lack of research or resources to aid professionals (e.g., educators, clinicians, physicians, case managers) in supporting autistic sexuality and relationships was also emphasized. Group 1 included six themes with four, described below, in the top ten. Themes are described in their ranked order, based on the values attributed by participants, within their thematic grouping.

#### *How to support healthy and satisfying romantic relationships? (Priority Ranking #1)*

Participants identified the need to support autistic people who desire a relationship in initiating and sustaining healthy relationships. Research is needed on ways people can develop realistic expectations about interactions in romantic relationships, and gain knowledge and skills such as initiating contact with a potential partner, and dating skills (e.g., where to find a partner, meet new people, and learning how to flirt). Also, questions were raised on how autistic people can (learn to) communicate about needs and desires and increase mutual understanding between partners. Attention was drawn to agency (e.g., what can help to take initiative, to know what to do and when to do it, dealing with implicit rules and expectations), self‐management (e.g., how to handle emotions and arousal, how to recognize and leave destructive relationships, how to navigate external norms and expectations, how to keep a balance between being together and keeping time for yourself), and how to disclose one's autistic identity in a relationship.

#### *How to support and promote sexual well‐being? (Priority Ranking #2)*

The need for additional evidence on how to deliver developmentally appropriate comprehensive sexuality and relationship education to autistic youth and adults using evidence‐based practices was stressed. Attention is warranted on how to align with a person's cognitive and emotional development in terms of topics, depth, and instructional strategies, with a focus on knowledge, skills, attitudes and identity development. In addition to autistic individuals, families and professionals may also need guidance on how to discuss and provide education on sexuality (e.g., how to communicate effectively and best developmental timing of topics). Individuals also learn from their own sexual experiences; the role and way of discussing and evaluating these experiences (e.g., with professionals) should be further explored.

#### *How to prevent sexual victimization and sexual offending? (Priority Ranking #4)*

Autistic youth and adults are at increased risk for sexual victimization compared to people in the general population and these experiences can undermine an individual's sexual, psychological, and physical health. Especially non‐autistic participants called for research on preventing sexual victimization, the need to modify interventions for sexual trauma to the needs of autistic people, and for research on preventing sexual (re)offending perpetrated by autistic people through the use of evidence‐based interventions. In the autistic groups, this theme was ranked lower compared to the total group of participants.

#### *How to involve parents, partners, and professionals to support the sexual well‐being and relationship satisfaction of autistic people? (Priority Ranking #5)*

Parents, spouses, other family members, and different types of professionals play an important role in sexuality education and support. Participants proposed the possibility of creating guidelines, trainings, and other resources for families and professionals, and to evaluate their impact. Non‐autistic stakeholders raised questions about their possible roles in supporting autistic relatives. Autistic participants stressed the importance for professionals to listen to and learn from the experience of autistic people. A recurring theme was that professionals need to be trained to initiate discussion and to provide effective support about sexuality and relationships with autistic people.

Other themes in this thematic grouping that were not ranked among the top ten themes included *sexual and reproductive healthcare needs and experiences* (Priority Ranking #13) and *the influence of pharmacological treatment on sexual functioning* (Priority Ranking #17).

### *Group 2: A Comprehensive Understanding of Sexuality and Relationships*

Participants identified gaining a comprehensive understanding of sexuality development and the life course sexual and relationship experiences of autistic adolescents and adults as a second group of priorities. Research on similarities and differences in sexuality between autistic and non‐autistic people can offer guidance or serve as a reference for autistic people to understand their own experiences and needs and could also be helpful for families and professionals in support roles. Attention to diversity was emphasized in terms of the degree and targets of sexual attraction, gender and sexual identities, different cultural contexts, and levels of intellectual functioning. Group 2 included nine themes, and six of them, described below, appeared in the top ten highest ranked priorities.

#### *How do autistic adolescents and adults experience and navigate sexuality across the lifespan? (Priority Ranking #3)*

Participants identified that a high priority is adopting a life course approach and investigating how sexuality development can differ within autistic individuals, and between autistic and non‐autistic individuals. Stakeholders emphasized that sexuality development begins in childhood and is important for adolescents and adults of all ages, including the elderly. Longitudinal research on sexuality development can allow researchers to study developmental pathways through which early experiences (e.g., early friendships, bullying and exclusion, and early positive or negative sexual experiences) influence adult sexuality and relationships.

#### *How does sexual and gender identity develop in autism? (Priority Ranking #6)*

Given the greater variation in gender and sexual identity that has been found in clinical and convenience samples of autistic youth and adults, participants identified gender and sexual identity developmental processes as another priority for research. Participants were especially interested in social construction approaches to understanding identity development, with questions centered on how autistic people develop and refine multiple intersecting identities. For example, common questions were whether more autistic people report gender variance and nonheterosexual identities, or how people combine different intersecting identity dimensions (gender identity, sexual identity, and autistic identity). Another important avenue of investigation is how societal attitudes and beliefs, for instance expressed in media portrayals and sex education, affect the way that autistic individuals and their families define and affirm gender and sexual identities.

#### *What is unique about autistic sexuality and intimacy? (Priority Ranking #7—Tied)*

Another theme of questions was how autism and related information processing characteristics may influence sexuality and relationships. Information processing included sensory sensitivity, self‐regulation, strong interests, use and interpretation of language, communication, rigidity, and theory of mind capabilities. These were thought to influence sexual interest, whether people were able to initiate dates and optimal ways of doing so, the development of long‐term intimate partnerships, and (mutual) responsivity to a partner during sexual activities. Participants were particularly interested in guidance about managing these information processing characteristics in sexual or romantic interactions.

#### *What works for whom in relationships? (Priority Ranking #7—Tied)*

Although Western cultures tend to promote long‐term, monogamous, legally binding partnerships that produce children as the goal for citizens, there are many different relationship configurations. Questions were raised about relationship satisfaction in autistic versus “mixed neurotype” couples (e.g., those in which one person is autistic and another is not), and in configurations allowing for more privacy and alone time or less stimulation (e.g., living apart together in which committed partners live in separate places). Also, attention was raised to the role and development of expectations by autistic people toward a relationship and toward dating: for instance, what people look for in their partners, what people expect while dating, and how to adjust expectations in a relationship.

#### *What are the experiences and needs of LGBT+ identifying autistic individuals? (Priority Ranking #9)*

LGBT+ identifying or questioning autistic individuals might face specific challenges, such as coming out twice (as LGBT+ and autistic). Further implications of living under the “double rainbow,” not feeling related to the visible LGBT+ community, and dealing with reactions from others were stressed. Participants called for attention to the experiences of LGBT+ identifying individuals. During the group discussions, participants also indicated the need for joining groups with peers to share experiences.

#### *How do sexuality and relationships sevelop in autistic individuals with lower levels of intellectual functioning? (Priority Ranking #10—Tied)*

The need for additional insight into sexuality development, relationship needs, and related experiences of autistic individuals with intellectual disabilities was stressed. Most themes referred broadly to the need to include people with intellectual disabilities in research on sexuality and relationships rather than providing specific questions of interest. No autistic participants with an intellectual disability participated in the present study, which could have resulted in more specific questions and a higher priority rating.

Other themes in this thematic grouping that were not ranked among the top ten themes included *how are relationships and sexuality related to health and well‐being* (Priority Ranking #12), *pregnancy and parenthood* (Priority Ranking #15—tied), and *how cultural context affects sexuality and relationships* (Priority Ranking #14).

### *Group 3: Autistic Perspectives in Research, Healthcare, and Society (Priority Ranking #10—Tied)*

Participants stressed not only the importance of directly influencing the lives of autistic people; they also explicitly referred to the importance of changing societal views on autistic sexuality. Research can challenge stereotypical views and stigmatization, raise awareness about the centrality of sexual well‐being to health and general well‐being, bring attention to diversity, and can translate findings into policy and guidelines that will improve quality of life for the autistic community. The need for participatory research was stressed, with involvement of and collaboration with autistic individuals in research (Priority Ranking #15—tied) in order to strengthen the relevance of studies to autistic people, and in turn also influence the views of parents, professionals, and society. Apart from questions for future research, these were clear statements on the way research‐projects should be organized and on the importance of disseminating knowledge broadly back to the community.

## Discussion

This *Commentary* reflects the ambition of the 2018–2020 INSAR Gender, Sexuality, and Romantic Relationships SIG to promote research on topics that are of high priority to autistic people and other stakeholders and which have a strong potential to improve health and quality of life. We used a consensus methodology to define research priorities using input from a variety of stakeholders. Based on the results, research is needed on ways to support sexual health, sexual well‐being and relationship satisfaction, and on insight in life course development of sexuality and relationships in a manner that is responsive to the diversity of the autism population. The high rankings associated with research on support for sexual health and sexual well‐being (e.g., prevention programs, educational interventions, service system improvements) showed this to be a clear priority for all stakeholders. A better understanding of the experiences and needs of autistic individuals during the lifespan can contribute to more accurate and nuanced views by parents, professionals, and community members on sexuality and relationships, can offer a framework to autistic people to understand their own experiences, and can lead to more attuned education and support. Insight in sexuality development and sexual well‐being can directly influence education and support, and improve societal views on autism, sexuality, and relationships.

Offering an overview of the existing knowledge on autism, sexuality, and romantic relationships was outside the scope of this article. However, some of the questions raised in this paper have already been partly studied, such as those related to sexual experience in adolescents and adults, sexuality education, gender identity, and relationship experience. Reviews of published research are available [see Bertilsdotter Rosqvist, 2014; Dewinter, Vermeiren, Vanwesenbeeck, & van Nieuwenhuizen, 2013; Kellaher, 2015; Pecora, Mesibov, & Stokes, 2016; Solomon, Pantalone, & Faja, 2019; van der Miesen, Hurley, & de Vries, 2016]. However, limitations in the existing research (e.g., small samples, focus on intellectually able young adults, and lack of racial/ethnic or gender diversity) often hamper interpretation and application or warrant replication studies, such as studies on sexual experience in adolescence and adulthood. Research questions generated in this study also highlighted several important directions for future research including longitudinal research on sexuality development and functioning over the life course, qualitative studies on how autistic people experience sexuality and romantic relationships, development and evaluation of comprehensive sexuality education programs, and ways to support people in their sexual lives and relationships.

Participants stressed the importance of incorporating perspectives of autistic people and participatory research designs, which might accelerate that future studies lead to relevant results and more accurate views on sexuality and relationships in autistic people. Confronting the qualitative analysis with the existing literature, these themes are in line with proposals for a combined participatory and translational approach [Stahmer, Aranbarri, Drahota, & Rieth, 2017]. Translational science implies that basic science (understanding) and applied science (support) reciprocally influence one another such that clinical insights spark ideas that lead to new insights in basic science, which are developed into new clinical treatments, services or resources. A participatory approach can strengthen our understanding of the needs of autistic individuals [Kapp, Goldknopf, Brooks, Kofner, & Hossain, 2019; Strang et al., 2019] and ensure that our basic research and applications improve the quality of life of autistic individuals.

While the use of NGT is a strength of this project, the resulting research priorities should also be interpreted in the context of this study's limitations. First, the prioritization of themes is based on the input of a relatively small number of participants. Although our goal was to involve participants from diverse backgrounds, most participants lived in Europe and North America, communicated verbally, and had the resources to attend a conference, so it is likely that important stakeholder groups were underrepresented or not included. Furthermore, to increase participant comfort, limited demographic information was collected, making it difficult to identify gaps in the generalizability of our results. Based on our experiences and the state of research in the field, we would argue for greater attention to the experiences of individuals from non‐Western cultures, middle age and older adults, people functioning below the average intelligence range, and to those who use minimal or no verbal language to communicate. A larger and more diverse group might have resulted in different priorities. Second, as noted by participants, it is important to include stakeholders in all stages of the research process in order to enhance the relevance of the project to the daily lives of autistic people with diverse backgrounds and characteristics. We acknowledge that this study did not include participation at all stages of the research process (i.e., during the thematic analysis of topics). Thus, this commentary offers an emerging agenda to guide future research and an example of a participatory study and will benefit from updates with a more diverse group of stakeholders. Despite these limitations, the discussion of research priorities during this project offered insight in how the participating autistic individuals experience their sexuality, and resulted in supportive conversations, sharing advice, and exchanging ideas about sexuality and relationships between autistic adults, professionals, and researchers. The questions and ideas in this paper are provided to guide and inspire researchers in designing future studies, not only pertaining to their research questions, but also to their approach and collaboration with autistic people.

## Compliance with Ethical Standards

All procedures performed in studies involving human participants were in accordance with the ethical standards of the institutional and/or national research committee and with the 1964 Helsinki declaration and its later amendments or comparable ethical standards. This study was approved by the School of Social and Behavioral Sciences Ethics Review Board at Tilburg University (EC2018.44).
